# Sustainable and Environmentally Friendly Na and Mg Aqueous Hybrid Batteries Using Na and K Birnessites

**DOI:** 10.3390/molecules25040924

**Published:** 2020-02-19

**Authors:** Francisco Gálvez, Marta Cabello, Pedro Lavela, Gregorio F. Ortiz, José L. Tirado

**Affiliations:** Departamento de Química Inorgánica e Ingeniería Química, Instituto Universitario de Química Fina y Nanoquímica, Universidad de Córdoba, Edificio Marie Curie, Campus de Rabanales, 14071 Córdoba, Spainiq1ticoj@uco.es (J.L.T.)

**Keywords:** sodium and potassium birnessite, hybrid magnesium batteries, aqueous electrolyte

## Abstract

Sodium and magnesium batteries with intercalation electrodes are currently alternatives of great interest to lithium in stationary applications, such as distribution networks or renewable energies. Hydrated laminar oxides such as birnessites are an attractive cathode material for these batteries. Sodium and potassium birnessite samples have been synthesized by thermal and hydrothermal oxidation methods. Hybrid electrochemical cells have been built using potassium birnessite in aqueous sodium electrolyte, when starting in discharge and with a capacity slightly higher than 70 mA h g^−1^. Hydrothermal synthesis generally shows slightly poorer electrochemical behavior than their thermal counterparts in both sodium and potassium batteries. The study on hybrid electrolytes has resulted in the successful galvanostatic cycling of both sodium birnessite and potassium birnessite in aqueous magnesium electrolyte, with maximum capacities of 85 and 50 mA h g^−1^, respectively.

## 1. Introduction

The current model of socio-economic development is based on the consumption of various energy resources. Fossil fuels have two main problems: Their reserves are finite and are close to depleted, and their use involves the generation of polluting compounds that affect the environment in numerous ways, leading to climate change. Renewable energies, such as wind and solar, with a more sustainable nature and more friendly with the environment, have the disadvantage of being intermittent. Energy storage systems are therefore needed for their use [1,2,3,4]. The widespread use of Li-ion batteries that provide high voltage, and reduced weight also presents a number of drawbacks such as the use of contaminant non-aqueous solvents, the irregular geographical distribution and low abundance of lithium in the Earth’s crust, the toxicity of certain components such as cobalt, and the relatively low safety of lithium-ion systems by flammable and explosive organic solvents, especially if lithium is deposited on the anode. That is why the central focus of many recent studies [5,6,7,8] is to find a substitute for lithium, close energy characteristics, and a more sustainable character. The elements with energy characteristics closest to lithium are sodium [5,6], magnesium [7] and potassium [8]. 

Birnessites are inorganic solids with a laminar structure formed by manganese oxide: MnO_6_ octahedrons forming sheets by sharing edges, which are stacked leaving an interlaminar space, a fact that allows this compound to possess an excellent ability for ion exchange. The interlaminar space can be occupied by water and cations.

**Figure 1:**
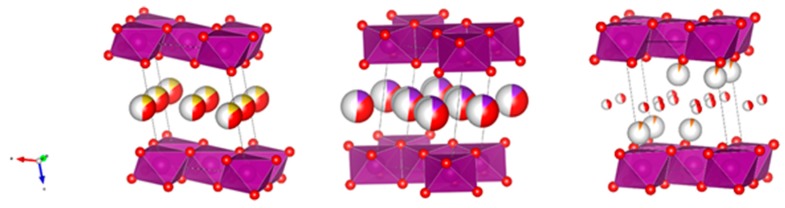
Structures of sodium, potassium and magnesium birnessites, respectively (violet: manganese, red: oxygen, yellow: sodium, indigo: potassium, and orange: magnesium). The large spheres with red regions indicate the presence of water together with the corresponding cations in the interlayer space.

In this work, two types of birnessite have been synthesized: sodium birnessite (Na-B) and potassium birnessite (K-B). Magnesium intercalation (Mg-B) has also been attempted. All three structures are known [9,10]. Figure 1 shows projections of the three structures, making use of known crystallographic data [9,10] and the VESTA program [11]. It should be noted that water molecules and sodium and potassium cations appear at the same height in the corresponding interlayers, while magnesium is placed in two layers closer to manganese. According to the bibliography data, the basal spacing changes significantly with the cation: 7.1408(3) Å for Na-B [10], 7.0865(1) Å for K-B [10], and 7.0068(3) Å for Mg-B [9].

Birnessites have been studied as electrode materials in sodium [12,13], potassium [14] and magnesium batteries with both aqueous and non-aqueous electrolyte [15,16]. In these studies, the alkali birnessite corresponding to the alkali electrolyte salt is always used. In aqueous electrolytes, Mg^2+^ hydration decreases the penalty of interfacial energy by allowing the Mg-B to achieve a large reversible capacity close to 230 mA h g^−1^ at an operating voltage of 2.8 V vs. Mg/Mg^2+^, showing the importance of an effective protection of the solid host and easy transfer of Mg^2+^ ions through the cathode interface upon charge and discharge [15]. Regarding hybrid electrolytes, it should be noted that the possibility of a calcium electrolyte versus potassium birnessite has already been examined [17]. Walter et al. [18] argued that, in order to fully exploit the benefits of a moderately priced metal Mg anode through hybrid battery concepts, it is desirable to combine the electrolyte with low-cost sodium, rather than lithium. According to Li et al. [19], the Na-Mg hybrid design could integrate the advantages of Mg metal anode and Na-ion intercalation cathode to improve the safety, rate capability and cycling stability. In addition, Bian et al. [20] found high efficacy for Na^+^/Mg^2+^ co-intercalation and suggested that the specific energy and volumetric energy of dual electrolyte batteries could potentially double.

The main objective of our study is to apply the concept of hybrid aqueous electrolyte in three novel electrochemical cell assemblies, containing alkali birnessites as not considered in the literature: K-B vs. Na, Na-B vs. Mg, and K-B versus Mg with the intention of improving the electrochemical behavior, as has already been found in other systems. The samples prepared by the solid-state and hydrothermal synthesis will be henceforth referred to as A-B/SS and A-B/HT (A: Na, K). 

## 2. Results and Discussion

**Figure 2:**
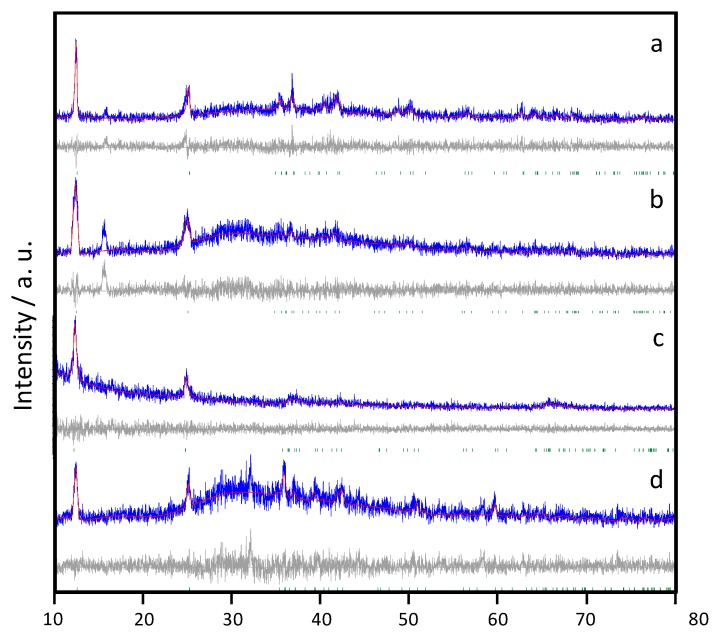
Powder XRD patterns of as-prepared birnessite samples: (**a**) Na-B/SS, (**b**) Na-B/HT, (**c**) K-B/SS, (**d**) K-B/HT. Blue: experimental profile, red: calculated profile, grey: difference profile, grey lines: peak positions. The CIF files provided in [10] were used as reference. This file is available in the Crystallography Open Database (COD), http://www.crystallography.net/. The original data for this entry were provided by the American Mineralogist Crystal Structure Database, http://rruff.geo.arizona.edu/AMS/amcsd.php. The file may be used within the scientific community so long as proper attribution is given to the journal article from which the data were obtained.

The structure of freshly prepared samples was examined by X-ray diffraction. The diffractograms shown in Figure 2 reveal the presence of birnessites indexable in the *C-1* space group of the triclinic system, as reported elsewhere [10], and were fitted according to the Le Bail method. An additional peak at ca. 15° (2θ) was found in Na containing birnessites, which could be associated to the presence of Na_0.7_MnO_2.05_ (ICCD #27-0751). This peak appears more remarkable for Na-B/HT. Concerning K-birnessites, the solid-state process yielded a pure sample indexed in the *C-1* space group. Similar to Na-birnessites, the hydrothermal route involved more impurities. In the latter case, a peak at ca. 32° (2θ) with significant contribution was ascribable to Mn_3_O_4_ (ICCD #16-0154). 

The studied birnessites were characterized by a limited number of intense diffraction peaks at low angles, which correspond mainly to the (00l) reflections. This is the result of two phenomena. On the one hand, the low crystallinity of products obtained without hydrothermal growth causes the intensities to be reduced. This is the result of two phenomena. On the one hand, the low crystallinity of products obtained without hydrothermal growth causes the intensities to be reduced. Moreover, the laminar character of the structures (Figure 2) would favor the preferential orientation of the particles, with the consequent relative increase in basal reflections. 

The spacing values between two successive sheets is defined by:

l·*d*_001_ = *c* sin β (1)

where *c* is one of the dimensions of the unit cell, and β the angle between the *c*-axis and the *ab* plane, parallel to the layers. From the experimental *d*_00l_ spacing values the basal spacing values can be calculated, which indicates the distance between successive sheets. The obtained values, 7.123(6) Å for Na-B and 7.089(3) Å for K-B, are in accordance with the literature [9,10]. 

**Figure 3:**
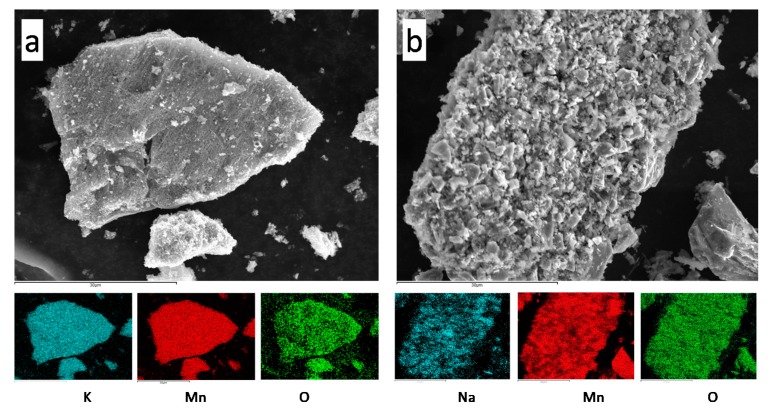
FESEM images and composition maps of (**a**) K-B/SS, (**b**) Na-B/SS.

Figure 3 shows the FESEM micrographs of samples K-B/SS and Na-B/SS. At low magnification, large aggregates of primary particles of several tens of micrometers and complex texture are observed. At higher resolution, primary particles of various microns in diameter and smooth surface can be seen, especially for Na-B/SS, which would be in accordance with particles exfoliated according to the direction perpendicular to [001], associated with the laminar character of the birnessite structure.

The electrochemical characterization was performed, as already mentioned, using three-electrode cells, the potential of the Ag/AgCl electrode as a reference, and a constant current density of C/8 for sodium and C/10 for potassium containing birnessites. According to the literature, it is known that the oxidation state of manganese in sodium and potassium birnessite takes approximate values of +3.42 [13] and +3.33 [14], with stoichiometries close to Na_0.58_MnO_2_·0.5H_2_O and K_0.77_MnO_2_·0.5H_2_O, respectively. In addition, the structure of both phases (Figure 2) shows a partial filling of the interlaminar space by alkaline cations. Accordingly, it is expected that manganese could be oxidized up to +4.00 by full alkali cation extraction during a first cell charge. Also, starting with the discharge of the battery, it would be possible to insert the alkali cation until an AMnO_2_ stoichiometry (A-Na and/or K) is completed. Thus, the profiles of the galvanostatic cycles of the various cells of our study, using both initial charge and discharge are discussed below.

**Figure 4:**
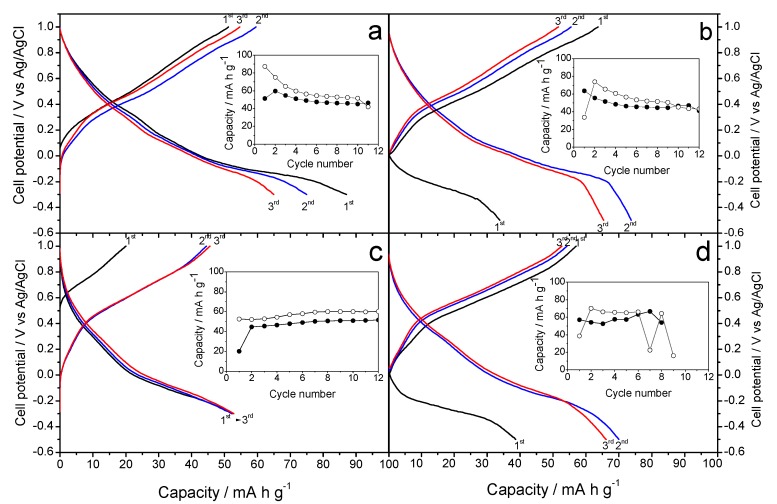
Galvanostatic curves in aqueous Na_2_SO_4_-based electrolyte and referring the voltages to the Ag/AgCl electrode recorded at C/8: (**a**,**b**) Na-B/SS starting with (**a**) charge and (**b**) starting with discharge (**c**,**d**) Na-B/HT starting with (**c**) charge and (**d**) starting with discharge. Insets: capacity vs. cycle number (open circles: discharge, filled circles: charge).

Figure 4a,b show the galvanostatic curves resulting when the Na-B (Na-B/SS and Na-B/HT) sample is used as a cathode material in aqueous Na_2_SO_4_ electrolyte. Figure 4a reveals a first charge profile, which is similar to that reported by Zhang et al. [13]. However, some differences can be discerned from the results of those authors, possibly due to the different kinetics used. Thus, the average voltage in our experiment takes a slightly lower value of 0.40 V, and no significant differences are observed between the first and successive charge branches. The differences between the first charge and discharge agree well with the initial stoichiometry. The capacity of the first discharge is 90 mA h g^−1^, higher than in reference [13], probably as a result of the slower kinetics that get closer to OCV values. However, the capacity decreases during the next cycles, and eventually stabilizes by about 50 mA h g^−1^ (inset in Figure 4a).

**Figure 5:**
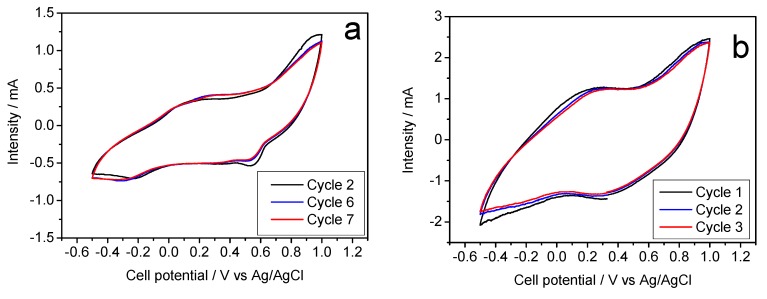
Cyclic voltammetry in aqueous Na_2_SO_4_-based electrolyte and referring the voltages to the Ag/AgCl electrode: (**a**) Na-B/SS starting with (**b**) K-B/SS.

Alternatively, a second galvanostatic experiment was scheduled to initiate by discharging the cell (Figure 4b). This situation results in a marked difference between the first and successive discharges, which would be justified again by the expected stoichiometry of the Na-B/SS. However, the average voltage was similar to that of Figure 4a, as expected from an analogous electrochemical process. The plotting of charge and discharge capacities (inset in Figure 4b) shows that the initial values are somewhat lower than those of Figure 4a, although again it stabilizes at about 50 mA h g^−1^. Finally, the cyclic voltammetry of Na-B/SS in sodium half-cells (Figure 5a) is in good agreement to the galvanostatic cycles. Thus, the absence of horizontal plateaus in the latter experiments results in poorly defined voltammetry peaks. However, the potential values of these peaks match the oxidation and reduction voltages and lead to an average of about 0.40 V mentioned above.

**Figure 6:**
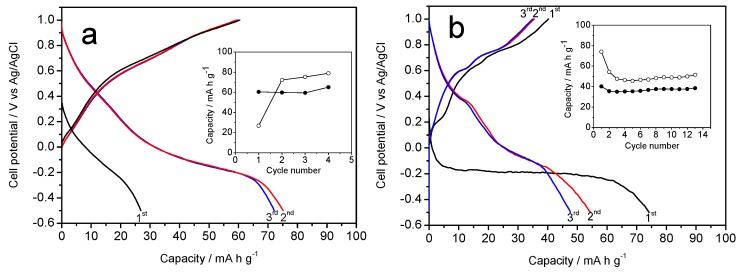
Galvanostatic curves in aqueous Na_2_SO_4_-based electrolyte recorded at C/10, referring the voltages to the Ag/AgCl electrode and starting with discharge: (**a**) K-B/SS and (**b**) K-B/HT. Insets: capacity vs. cycle number (open circles: discharge, filled circles: charge).

Figure 4c,d show the galvanostatic curves for Na-B/HT sample. In this case, we can see significant differences with Na-B/SS. Figure 6a,c have maximum capacities of 50 and 30 mA h g^−1^ respectively, having a large difference in capacity if we start in charge (Figure 4c) or discharge (Figure 4d); thus differing from the experiences described by Zhang et al. [13]. As with the Na-B/HT, the average voltage value around 0.40 V, with no apparent differences between successive galvanostatic curves. The inset in Figure 4c shows that the capacity remains stable slightly higher than 50 mA h g^−1^, ascending slightly with each cycle; differing from the capacity shown in Figure 4d, which has a clear instability. These experiments give little interest to further characterize electrochemically the Na-B/HT sample.

Figure 6a shows the galvanostatic profiles recorded for the K-B/SS sample as a cathode material in sodium-ion aqueous Na_2_SO_4_-based electrolyte. These experiments can be indeed described as a hybrid aqueous electrolyte. Potassium birnessite has been studied in detail by Lin et al. [14], in potassium half-cells and non-aqueous electrolyte of KPF_6_ in ethylene carbonate/diethyl carbonate. Unlike this study, Figure 6a and b show alternative results, since the electrolyte is Na_2_SO_4_, thus being the first electrochemical cell consisting of an aqueous Na/K hybrid electrolyte and a potassium birnessite as cathode. Unlike to the study using a K/Ca hybrid electrolyte, as reported by Hyoung et al. [17], the capacity appears to have an upward trend, starting with a value of 70 mA h g^−1^ and rises 5 points in each of the following cycles. The average voltage is 0.4 V, similar to previous and subsequent experiments. The cyclic voltammetry (Figure 5b) now shows defined signals, coinciding with the aforementioned average voltage of the galvanostatic plateaus.

Under the same operating conditions as in the previous experiments, Figure 6b shows the galvanostatic profile resulting from the use of the K-B/HT sample as a cathode material. The irregular character of the curve is noteworthy, with stable capacities approximately 50 mA h g^−1^ in discharge and about 40 mA h g^−1^ in charge. Figure 6b shows a stable profile, Compared to K-B/SS, this hydrothermal synthesis does not involve improvements of performance that would be worthy of mention. For this reason, the voltammetry study was ruled out.

After observing the behavior of the different samples against the Na electrolyte, new cells were mounted with Mg electrolyte. As mentioned above, the use of the magnesium electrolyte is regarded in the literature by Nam et al. [15] and Sun et al. [16]. In fact, the latter study also focused on the use of aqueous electrolyte.

**Figure 7:**
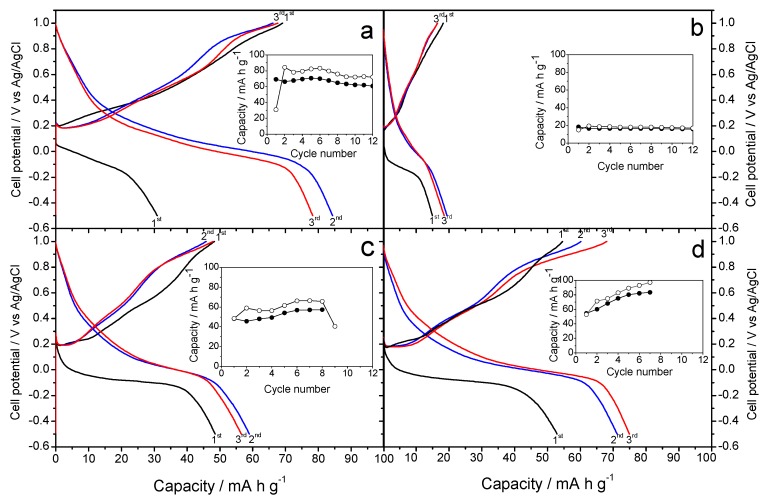
Galvanostatic curves in aqueous MgSO_4_-based electrolyte recorded at C/8, referring the voltages to the Ag/AgCl electrode and starting with discharge: (**a**) Na-B/SS (**b**) Na-B/HT (**c**) K-B/SS (**d**) K-B/HT. Insets: capacity vs. cycle number (open circles: discharge, filled circles: charge).

**Figure 8:**
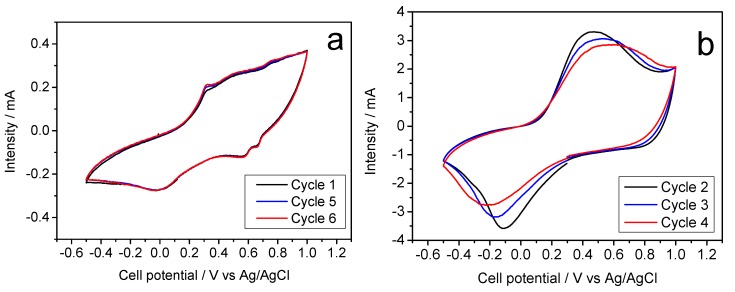
Cyclic voltammetry in aqueous MgSO_4_-based electrolyte and referring the voltages to the Ag/AgCl electrode: (**a**) Na-B/SS and (**b**) K-B/SS.

Figure 7. shows the galvanostatic curves of cells assembled with Na-B/SS as a cathode material in an aqueous MgSO_4_ electrolyte. This plot is the result of the study of sodium half-cell in a hybrid electrolyte. As in previous studies against hybrid electrolytes, as is the case with K cells, our working cathode, the Na-B/SS begins its discharge cycle. We can observe an average voltage of about 0.30 V, slightly less than 0.4 V recorded in our previous experiments with this material and the studies of Sun et al. with the Mg [16], being more like those of Nam et al. [15]. A gradual loss of capacity is observed as the cycling progresses, descending from approximately 85 to near 50 mA h g^−1^, well below those reported in [12,13]. In turn, the cyclic voltammogram recorded for this sample reveals poorly defined voltammetry peaks which intensity can hardly reach 0.5 mA, Similarly, the experience with the Na electrolyte (Figure 8a). For Na-B/HT, Figure 7b shows its good cyclability though a low capacity value of only 20 mA h g^−1^ was recorded and hence further studies were ruled out.

Concerning of potassium birnessites as cathodes in hybrid magnesium half-cells, likewise to the study of K-B/SS in Na electrolyte, we can establish referential similarities. Thus, the studies of Nam et al. [15] and Sun et al. [16] are useful for comparing experiences regarding the behavior of the Mg electrolyte and the studies of Lin et al. [14] and those of Hyoung et al. [17] for the comparison of the behavior of K-B/SS, either the study of their general behavior or their behavior in a hybrid electrolyte. Figure 7c shows an average voltage of 0.3 V, thus differentiating from that shown in the K-B/SS experience from Na. The capacity remains stable around 60 mA h g^−1^. Otherwise, the galvanostatic cycling of K-B/HT revealed a continuous increase of capacity from 50 to 100 mA h g^−1^. It involves an electrode activation during the first few cycles (Figure 7d). This better behavior of the hydrothermally prepared potassium birnessite was confirmed by cyclic voltammograms in Figure 8b, where the presence of well-defined anodic and cathodic bands evidences the reliability of the electrochemical magnesium reaction. Thus, Figure 8b shows the corresponding cyclic voltammetry, presenting a current intensity of 3 mA and a well-defined curve, being able to clearly see in it the processes of oxidation and reduction of the half-cell.

## 3. Materials and Methods

Sodium and potassium birnessites were prepared by two alternative routes: thermal and hydrothermal. For thermal Na birnessite (Na-B/SS), a mixture of solid precursors was prepared by grinding 2 g Na_2_CO_3_ and 2 g MnO_2_ in an agate ball mill at 300 rpm for 2 h. Then the mixture was heated at 850 °C for 10 h in air. The product was washed with deionized water several times and dried at 70 °C for 10 h in air. For the synthesis of K birnessite (K-B/SS), 1.71 g of KMnO_4_ were thermally decomposed in an alumina crucible at 350 °C for 10 h. The sample was then washed with deionized water and kept at 80 °C for 4 days [14]. 

Alternatively, both alkali birnessites were also prepared by a hydrothermal route. For hydrothermal Na birnessite (Na-B/HT), 0.09 mmol of Mn_3_O_4_ were added to 100 mL of a 6 M NaOH aqueous solution in a Teflon vessel (Parr Instrument Company, Moline, IL, 61265 USA). This solution was then maintained in a hydrothermal vessel at 180 °C for 24 h in air. The solid product was then rinsed with deionized water several times and dried at 70 °C for 24 h in air. For hydrothermal K birnessite (K-B/HT), a similar procedure was followed by replacing NaOH by KOH solution.

Powder X-ray diffraction (XRD) patterns were recorded using a Bruker D8 Discover A25 diffractometer (Bruker Española S.A., Madrid, Spain), equipped with Cu Kα-radiation, Ge monochromator and Lynxeye detector with steps of 0.04° (2-theta). Particle morphology was examined using a JSM-7800F field-emission scanning electron microscope (FESEM, JEOL Ltd., Tokyo, Japan). 

The electrochemical experiments were carried out in three-electrode cells consisting of a working electrode composed by a mixture of the sample to be studied, carbon black and PVdF (polyvinylidene fluoride) in an 80:10:10 ratio. In order to ensure the electrode homogeneity, this mixture was dispersed in NMP (N-methyl pyrrolidone), spread onto a titanium foil and dried at 120 °C for 2 h. Platinum was used as counter-electrode and Ag/AgCl as reference electrode. Two different aqueous electrolytes: 1 M Na_2_SO_4_ and 1 M MgSO_4_ were used. Galvanostatic experiments were performed at several C rates in the potential window between −0.5 and 1.0 V vs. AgCl/Ag. Cyclic voltammetry was carried out at 0.2 mV s^−1^ in the same potential windows. Both experiments were controlled with a multichannel VMP potentiostat/galvanostat.

## 4. Conclusions

Sodium and potassium birnessite samples have been synthesized by alternative methods of air and hydrothermal oxidation. The highest purity was achieved by air. Sodium birnessite has been successfully cycled with aqueous sodium electrolyte, starting these cycles both in charge and discharge. Reversible capacity values stabilizing around 50 mA h g^−1^ have been obtained in both experiences. Hybrid electrochemical cells have been built using potassium birnessite in aqueous sodium electrolyte, when starting in discharge and with a capacity slightly higher than 70 mA h g^−1^. As expected by using a pure magnesium sulphate electrolyte, the first discharge implies magnesium intercalation, while charging above the first discharge would result in alkali ion deintercalation. The products of hydrothermal synthesis generally show slightly poorer electrochemical behavior than their air synthesis counterparts in both sodium and potassium batteries. The study on hybrid electrolytes has resulted in the successful utilization of sodium birnessite and potassium birnessite in the aqueous MgSO_4_ electrolyte, with a view on their potential application as the cathode and in low cost and environmentally friendly batteries using maximum capacities of 85 and 50 mA h g^−1^, respectively. 

## References

[1] Armand M., Tarascon J.-M. (2008). Building better batteries. Nature.

[2] Mauger A., Julien C.M., Paolella A., Armand M., Zaghib K. (2018). A comprehensive review of lithium salts and beyond for rechargeable batteries: Progress and perspectives. Mater. Sci. Eng. R.

[3] Mauger A., Julien C.M., Paolella A., Armand M., Zaghib K. (2019). Building Better Batteries in the Solid State: A Review. Materials.

[4] Mauger A., Julien C.M., Paolella A., Armand M., Zaghib K. (2019). Recent Progress on Organic Electrodes Materials for Rechargeable Batteries and Supercapacitors. Materials.

[5] Palomares V., Serras P., Villaluenga I., Hueso K.B., Carretero-Gonzalez J., Rojo T. (2012). Na-ion batteries, recent advances and present challenges to become low cost energy storage systems. Energy Environ. Sci..

[6] Xu G.L., Amine R., Abouimrane A., Che H., Dahbi M., Ma Z.F., Saadoune I., Alami J., Mattis W.L., Pan F. (2018). Challenges in Developing Electrodes, Electrolytes, and Diagnostics Tools to Understand and Advance Sodium-ion Batteries. Adv. Energy Mater..

[7] Gautam G.S., Canepa P., Malik R., Liu M., Persson K., Ceder G. (2015). First-principles evaluation of multi-valent cation into insertion orthorhombic V_2_O_5_. Chem. Commun..

[8] Vaalma C., Griffin G.A., Buchholz D., Passerini S. (2016). Non-Aqueous K-Ion Battery Based on Layered K_0.3_MnO_2_ and Hard Carbon/Carbon Black. J. Electrochem. Soc..

[9] Post J.E., Veblen D.R. (1990). Crystal structure determinations of synthetic sodium, magnesium, and potassium birnessite using TEM and the Rietveld method. Am. Mineral..

[10] Lopano C.L., Heaney P.J., Post J.E., Hanson J., Komarneni R. (2007). Time-resolved structural analysis of K- and Ba-exchange reactions with synthetic Na-birnessite using synchrotron X-ray diffraction. Am. Mineral..

[11] Momma K., Izumi F. (2011). VESTA 3 for three-dimensional visualization of crystal, volumetric and morphology data. J. Appl. Crystallogr..

[12] Xia H., Zhu X., Liu J., Liu Q., Lan S., Zhang Q., Liu X., Seo J.K., Chen T., Gu L. (2018). A monoclinic polymorph of sodium birnessite for ultrafast and ultrastable sodium ion storage. Nat. Commun..

[13] Zhang X., Hou Z., Li X., Liang J., Zhu Y., Qian Y. (2016). Na-Birnessite with High Capacity and Long Cycle Life Rechargeable Aqueous Sodium-ion Battery Cathode Electrodes. J. Mater. Chem. A.

[14] Lin B., Zhu X., Fang L., Liu X., Li S., Zhai T., Xue L., Guo Q., Xu J., Xia H. (2019). Birnessite Nanosheet Arrays with High K Content as a High-Capacity and Ultrastable Cathode for K-Ion Batteries. Adv. Mater..

[15] Nam K.W., Kim S., Lee S., Salama M., Shterenberg I., Gofer Y., Kim J., Yang E., Park C.S., Kim J. (2015). The High Performance of Crystal Water Containing Manganese Birnessite Cathodes for Magnesium Batteries. Nano Lett..

[16] Sun X., Duffort V., Mehdi B.L., Browning N.D., Nazar L.F. (2016). Investigation of the Mechanism of Mg Insertion in Birnessite in Nonaqueous and Aqueous Rechargeable Mg-Ion Batteries. Chem. Mater..

[17] Hyoung J., Heo J.W., Hong S. (2018). Investigation of electrochemical calcium-ion energy storage mechanism in potassium birnessite. J. Power Sources.

[18] Walter M., Kravchyk K.V., Ibanez M., Kovalenko M.V. (2015). Efficient and Inexpensive Sodium-Magnesium Hybrid Battery. Chem. Mater..

[19] Li Y., An Q., Cheng Y., Liang Y., Ren Y., Sun C., Dong H., Tang Z., Li G., Yao Y. (2017). A high-rechargeable magnesium-sodium hybrid battery. Nano Energy.

[20] Bian X., Gao Y., Fu Q., Indris S., Ju Y., Meng Y., Du F., Bramnik N., Ehrenberg H., Wei Y. (2017). A Long Cycle-Life and High Safety Na^+^/Mg^2+^ Hybrid-Ion Battery Built by using a TiS_2_ Derived Titanium Sulfide Cathode. J. Mater. Chem. A.

